# Clustering-independent analysis of genomic data using spectral simplicial theory

**DOI:** 10.1371/journal.pcbi.1007509

**Published:** 2019-11-22

**Authors:** Kiya W. Govek, Venkata S. Yamajala, Pablo G. Camara

**Affiliations:** Department of Genetics and Institute for Biomedical Informatics, Perelman School of Medicine, University of Pennsylvania, Philadelphia, PA, United States of America; University of California Davis, UNITED STATES

## Abstract

The prevailing paradigm for the analysis of biological data involves comparing groups of replicates from different conditions (e.g. control and treatment) to statistically infer features that discriminate them (e.g. differentially expressed genes). However, many situations in modern genomics such as single-cell omics experiments do not fit well into this paradigm because they lack true replicates. In such instances, spectral techniques could be used to rank features according to their degree of consistency with an underlying metric structure without the need to cluster samples. Here, we extend spectral methods for feature selection to abstract simplicial complexes and present a general framework for clustering-independent analysis. Combinatorial Laplacian scores take into account the topology spanned by the data and reduce to the ordinary Laplacian score when restricted to graphs. We demonstrate the utility of this framework with several applications to the analysis of gene expression and multi-modal genomic data. Specifically, we perform differential expression analysis in situations where samples cannot be grouped into distinct classes, and we disaggregate differentially expressed genes according to the topology of the expression space (e.g. alternative paths of differentiation). We also apply this formalism to identify genes with spatial patterns of expression using fluorescence in-situ hybridization data and to establish associations between genetic alterations and global expression patterns in large cross-sectional studies. Our results provide a unifying perspective on topological data analysis and manifold learning approaches to the analysis of large-scale biological datasets.

## Author summary

Manifold learning methods have emerged as a way of analyzing the large high-dimensional data sets that are currently generated in many areas of science. They assume the data has been sampled from an unknown manifold which is approximated with a graph and utilize spectral graph techniques to perform unsupervised feature selection and dimensionality reduction. However, graphs provide only partial approximations to manifolds, precluding the application to features with a complex combinatorial structure. Relatedly, these methods cannot take into account the topology of the manifold. In this work, we extend spectral methods for feature selection to topological spaces built from data and present a general framework for feature selection. We present specific applications of this framework to clustering-independent analysis of gene expression and multi-modal genomic data. In particular, using these methods, we perform differential expression analysis in situations where samples cannot be grouped into distinct classes, and we disaggregate the results according to topological features of the expression space. In addition, we identify genes with spatial patterns of expression using spatially-resolved transcriptomic data and establish associations between genetic alterations and global expression patterns in large cross-sectional cancer studies.

This is a *PLOS Computational Biology* Methods paper.

## Introduction

The standard paradigm for the design of biological experiments involves grouping samples and experiments into replicates from different conditions (for instance, control and treatment) and using the observed variability within replicates to assess the significance of the features that differ across conditions. The identification of features that discriminate samples from discrete and identifiable classes is a classical problem in statistics and data analysis. During the 20^th^ century, numerous non-parametric tests were developed to that end, including the Kolmogorov-Smirnov, Wilcoxon, and Mann-Whitney tests, which have become ubiquitous across all areas of science. In these problems, classes are inherent to the formulation of the problem or emerge from an underlying metric or semi-metric structure on the data which permits grouping samples into clusters and subsequently identifying differential features.

However, in modern genomics, there are many situations where replicates are not available. For example, true replicates are generally not possible in single-cell omics experiments as each cell is unique and typically can only be measured once. In those situations, the majority of the existing algorithms follow a two-step approach inspired by the above paradigm. First, samples are clustered based on some similarity measure; then, an analytic algorithm (e.g. differential expression analysis, spatial mapping, or bulk RNA-seq deconvolution) is applied to identify features that discriminate clusters, assuming the observed variability within each cluster is technical. This approach, however, has some important limitations. Very often samples cannot be naturally grouped into clusters, such as when they are drawn from a continuous process. Moreover, much of the variability within clusters is often caused by underlying dynamic and continuous biological processes (e.g. the cellular context in the case of single-cell omics data). In those situations, common tests of significance cannot be applied and feature selection becomes cumbersome.

**Fig 1:**
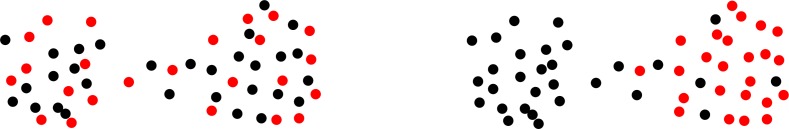
Variance can be used as a proxy for unsupervised feature selection but does not take into account the underlying metric structure of the data. In the figure a point cloud is labeled according to two binary features with equal variance. However, only the feature on the right shows a large degree of consistency with the metric structure of the data. Manifold learning approaches to feature selection prioritize features according to the degree of consistency with the underlying metric structure of the data.

A standard approach to feature selection when samples cannot be arranged into classes is to use variance as a proxy [1]. This approach assumes that features that have high variance across the entire data set are more informative about the structure of the data than features that take a constant value. Although the use of variance for feature selection has offered many benefits in multiple domains, it only makes use of the set structure and ignores the richer metric (or semi-metric) structure of the data when it is available (Fig 1). In this spirit, *manifold learning* methods, such as the Laplacian score [2] and diffusion maps [3], have become increasingly popular in the past decade, as technological progress has allowed generating the large amounts of high-dimensional vector-like data they require. These methods assume that each vector of measurements specifies the coordinates of a point in a high-dimensional manifold. They then construct a nearest neighbor graph to approximate the unknown manifold from the data and apply spectral graph techniques for dimensionality reduction and unsupervised feature selection. Spectral graph techniques have been utilized in genomics through spectral clustering [4, 5] and network-based regularization [6–8] algorithms. Additionally, manifold learning methods for dimensionality reduction, such as diffusion maps, have become very popular in the analysis of single-cell expression data [9, 10]. However, manifold learning methods for feature selection, such as the Laplacian score, have not been used to analyze genomic data.

**Fig 2:**
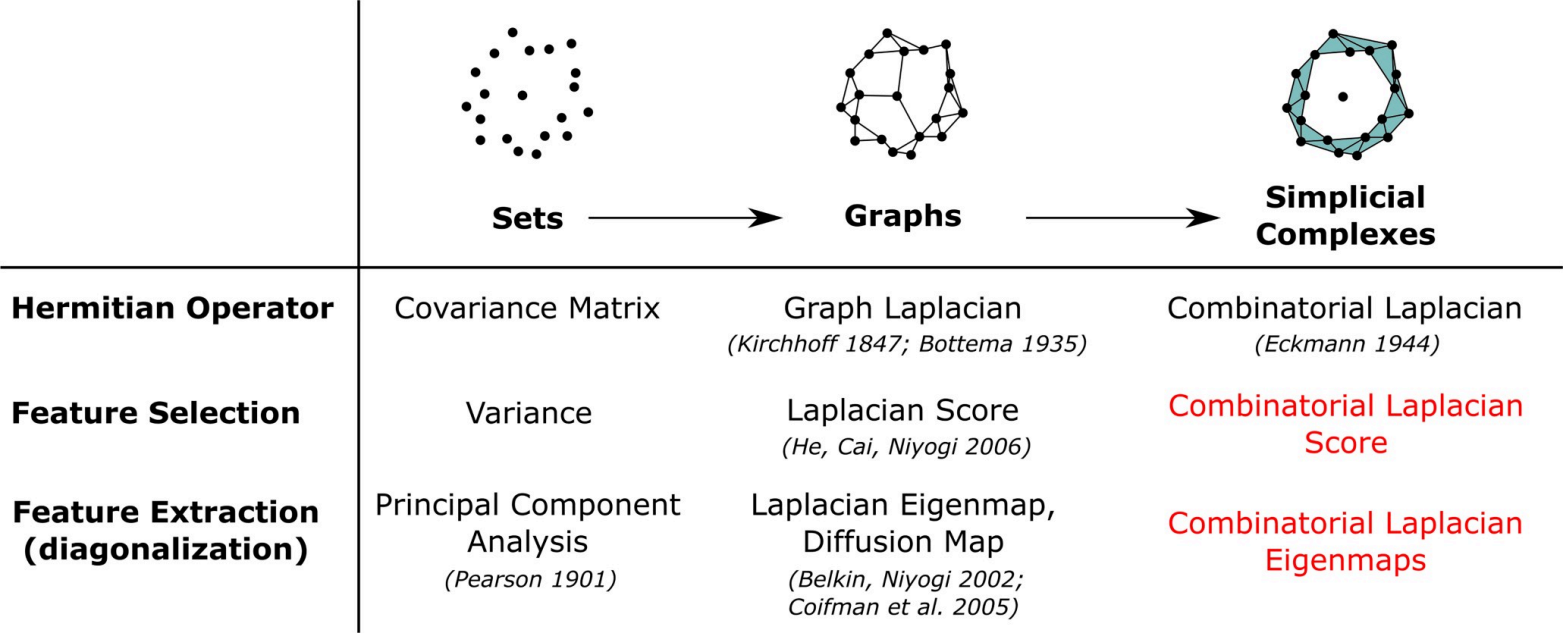
Summary of various related approaches to unsupervised feature selection and extraction, highlighting the concepts introduced in this paper.

In this paper, we introduce a general framework for unsupervised feature selection which extends current graph-based manifold learning methods (Fig 2) and we demonstrate its utility for the analysis of genomic data. Our framework builds upon simplicial complex representations of the data: algebraic generalizations of graphs that, apart from vertices and edges, can include higher-dimensional elements such as triangles and tetrahedrons. Simplicial complexes are a central concept in topology, geometry, and combinatorics, where they represent a powerful tool for the description and computation of topological spaces. Their use largely simplifies the study of complex relations of the data, such as loops induced by cell cycle effects or by alternative and convergent paths of differentiation [11], or cavities induced by multiple viral re-assortment [12], which are otherwise difficult to analyze using graphs.

We demonstrate the utility of this framework in the analysis of genomic data and present several applications where spectral simplicial methods lead to qualitatively different results compared to prevailing clustering-based methods. In particular, we use our framework to identify and disaggregate differentially expressed genes according to topological features of the expression space in situations where samples cannot be grouped into distinct clusters. We also show its utility for the analysis of multi-modal genomic data in two different applications: the identification of genes with spatial patterns of expression and the identification of associations between genetic alterations and global expression patterns in large cross-sectional studies. All of these applications represent novel uses of spectral methods in genomics and illustrate the potential of these methods over traditional clustering-based approaches in situations where samples cannot be naturally arranged in groups.

## Results

### Geometric feature selection

Genomic data sets can often be described as point clouds, which are collections of points with a notion of pairwise distance or dissimilarity, such that each point represents a sample. Common examples of pairwise dissimilarities used in genomics are correlation distance among gene expression profiles or genetic distances among genomic sequences. In this paper, we address the problem of unsupervised feature selection in point clouds. We define features of a point cloud as functions that assign a value to each sample. The expression level of a gene, the presence or absence of a mutation, or the methylation level of a gene promoter are some examples of point cloud features.

In 2006, He, Cai, and Niyogi proposed an algorithm for unsupervised feature selection called Laplacian score [2]. They construct a weighted nearest neighbor graph and introduce a score for each feature defined in terms of the graph Laplacian. The Laplacian score *R*_*r*_ ranks features according to their consistency with the structure of the nearest neighbor graph. Specifically, features with small values for *R*_*r*_ take high values in highly connected nodes of the graph. Although this approach to unsupervised feature selection is rarely used in the analysis of biological data, it has become widespread in other areas of data analysis, as it offers a substantial statistical power compared to ranking features according to their variance [2].

**Fig 3:**
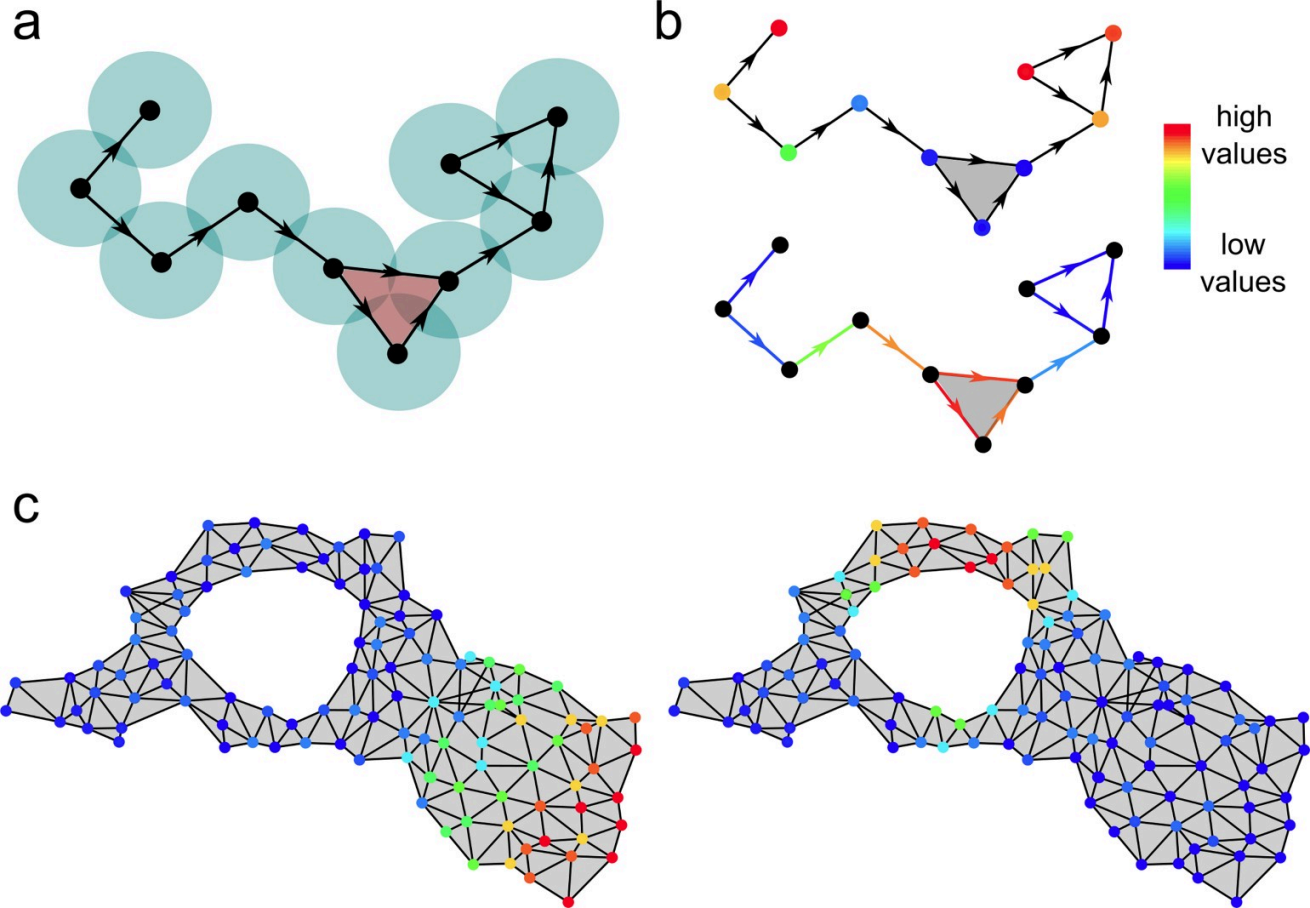
Simplicial complexes provide topological representations of a space. They are generalizations of graphs containing higher dimensional elements such as triangles, tetrahedra, etc. In some cases, higher dimensional elements in a simplicial complex can convey information of a point cloud that is not captured by the underlying graph. For example, in (a) a Čech complex is constructed from intersections of fixed-radius balls centered at the points of a point cloud, which in this example are ordered according to the horizontal coordinate. Simplicial complexes enable the application of co-homological techniques to point cloud features (that is, to functions defined over the elements of the point cloud). Features can be defined over individual points (b, top), pairs of points (b, bottom), triplets, etc. Co-homological techniques, like those discussed in this work, can rank and classify point cloud features according to their amount of localization along topological structures (disconnected components, loops, cavities, etc.) of the underlying simplicial complex. In (c), two examples of point cloud features localized along a topologically trivial region (left) or a non-contractible loop (right) are shown.

To be able to capture more complex relations of the data, we generalized the Laplacian score to simplicial complex representations of the data (S1 Note). Like graphs, simplicial complexes provide approximations to spaces from which a set of points have been randomly sampled. In particular, the skeleton spanned by the nodes and edges of a simplicial complex is a graph of the type considered in manifold learning approaches. However, simplicial complexes enable the study of arbitrarily general features in relation to the topological structure of the underlying space (Fig 3).

A simple approach to constructing a simplicial complex from a point cloud is building a Čech complex [13]. This is the complex that results from considering the set of balls with fixed radius centered at the points of the cloud and replacing the balls by nodes, the pairwise intersections between balls by edges, the triple intersections by triangles, etc. (Fig 3A). The utility of the Čech complex is ensured by the nerve theorem: if the point cloud was sampled from a topological space, under certain sampling assumptions the Čech complex approximates the topology of the space [13]. Since computing triple and higher-order intersections among balls is computationally costly, a simplification of the Čech complex known as the Vietoris-Rips complex is often used in practical applications (S1 Note). Vietoris-Rips complexes are a well-controlled approximation to Čech complexes in which all elements are determined by the pairwise intersections between balls [13], and are therefore faster to compute. Additionally, Uniform Manifold Approximation and Projection (UMAP) [14] and Mapper [15] are often used to generate reduced simplicial complex representations of the data that preserve much of the topology of Čech complexes.

**Fig 4:**
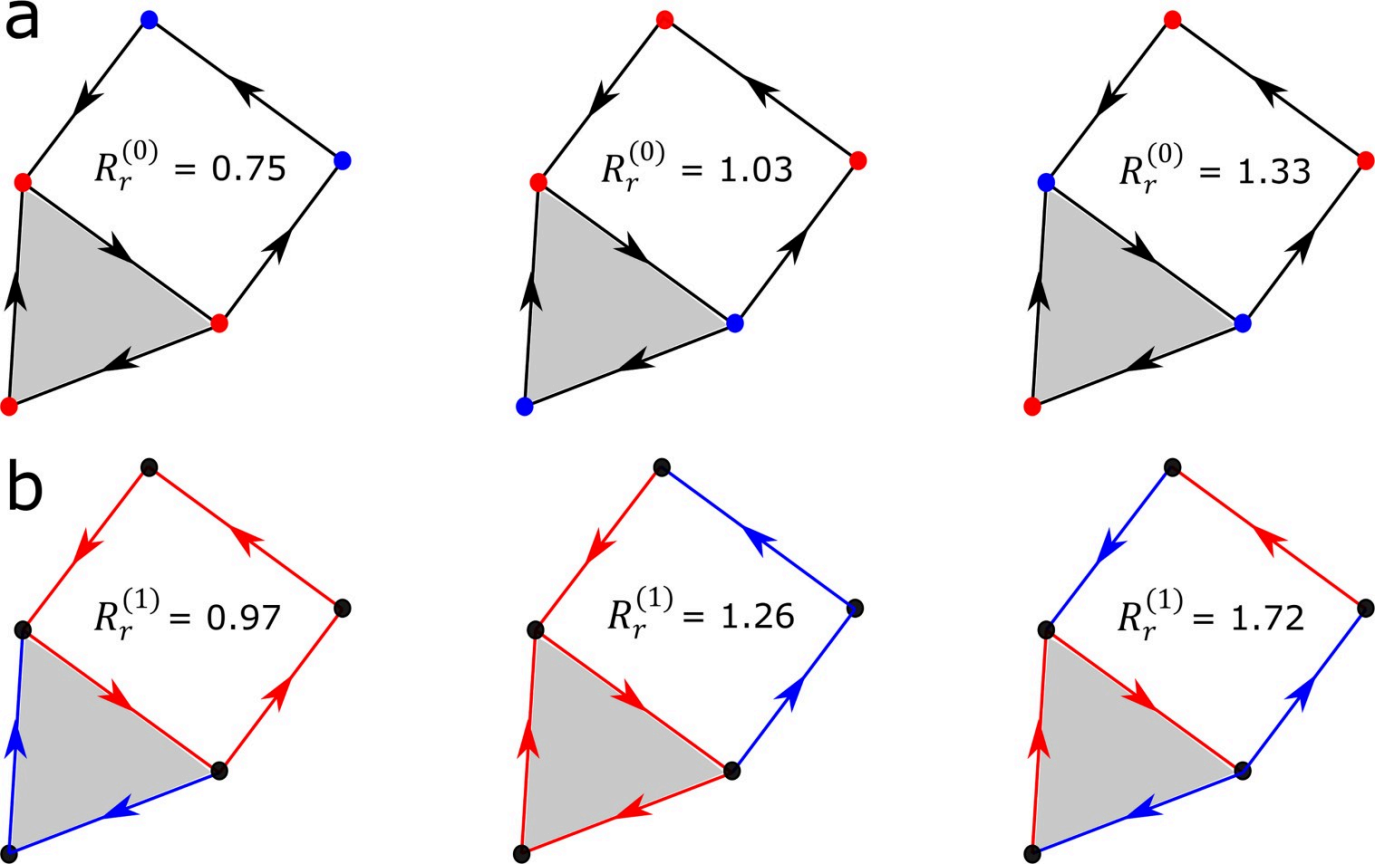
**Combinatorial Laplacian score for a set of binary 1-point (a) and 2-point (b) features on a Čech complex.** Features in this example can take values 0 (blue) or 1 (red). The 0-dimensional combinatorial Laplacian score $R_{r}^{\left(0\right)}$ was computed for each of the features shown in (a). These features assign values to the nodes in the complex. $R_{r}^{\left(0\right)}$ can be expressed as a sum over edges (S1 Note), where edges that connect vertices on which the feature takes a high value contribute little. Features that take high values on highly connected regions of the simplicial complex therefore have lower values of $R_{r}^{\left(0\right)}$ than features that take high values on disconnected nodes. Analogously, the 1-dimensional combinatorial Laplacian score $R_{r}^{\left(1\right)}$ was computed for each of the features shown in (b). In this case, features are evaluated on the edges of the simplicial complex. Features that take high values on edges that form non-contractible loops in the simplicial complex have lower values of $R_{r}^{\left(1\right)}$ than features that take high values on disconnected or contractible paths.

Building upon Eckmann’s generalization of the graph Laplacian to simplicial complexes [16], we introduced a family of combinatorial Laplacian scores $R_{r}^{\left(q\right)}$ that rank point cloud features according to their degree of consistency with the *q*-dimensional topology of a simplicial complex built from the data (S1 Note). In particular, $R_{r}^{\left(0\right)}$ corresponds to the ordinary Laplacian score on the graph skeleton of the simplicial complex and it ranks features according to their degree of localization in connected regions of the complex (Fig 4). Features that take high values in highly-connected regions of the complex (that is, regions for which most pairs of vertices are connected by an edge) have a low value of $R_{r}^{\left(0\right)}$. Similarly, $R_{r}^{\left(1\right)}$ takes low values for features that take high values along non-contractible loops spanned by the data (Fig 4); $R_{r}^{\left(2\right)}$ takes low values for features that take high values along cavities spanned by the data, etc. In summary, the combinatorial Laplacian score extends the ordinary Laplacian score for feature selection to higher-order relations of the data.

**Fig 5:**
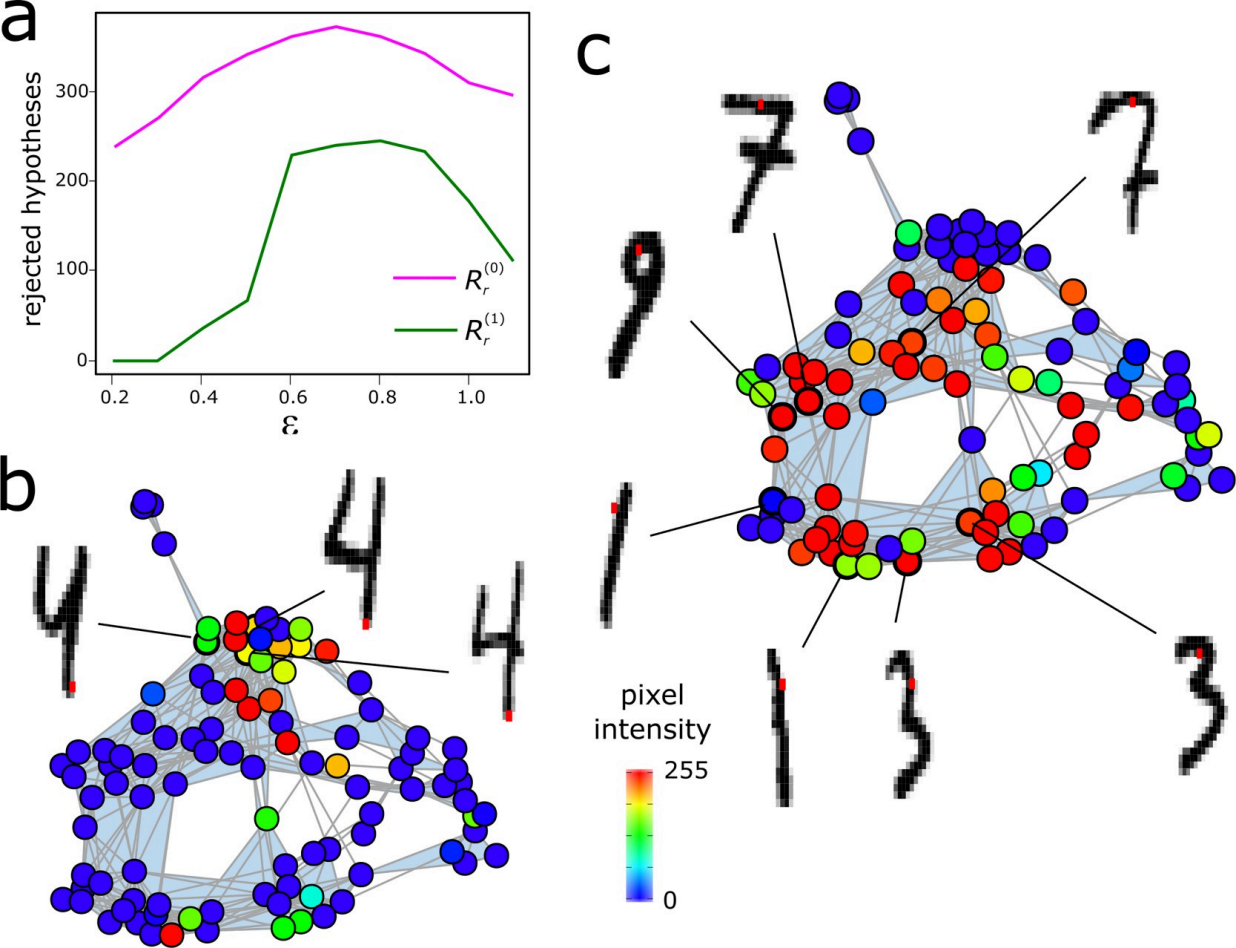
Feature selection on the MNIST dataset using the combinatorial Laplacian score. Each sample consists of a grey-scale image of a hand-written digit from 0 to 9 and each pixel represents a feature. The degree of localization of each feature in the simplicial complex is assessed using the 0- and 1-dimensional combinatorial Laplacian scores. (a) Number of rejected null hypothesis at a FDR of 0.05 as a function of the radius *ε* of the balls in the Vietoris-Rips complex. In this example, the statistical power of $R_{r}^{\left(0\right)}$ and $R_{r}^{\left(1\right)}$ is maximized for *ε*~0.7. The significance of the scores was determined through a permutation test were the pixels were randomized 5,000 times. (b) Vietoris-Rips complex colored by the intensity of a pixel that is significant under $R_{r}^{\left(0\right)}$ (q-value < 0.005) but not under $R_{r}^{\left(1\right)}$ (q-value = 0.5). The intensity of the pixel is high in a densely connected, topologically trivial region of the complex containing images of the digit `4`. Images associated to several nodes are shown for reference, with the pixel highlighted in red. (c) Vietoris-Rips complex colored by the intensity of a pixel that is significant under both $R_{r}^{\left(0\right)}$ (q-value < 0.005) and $R_{r}^{\left(1\right)}$ (q-value < 0.005). The intensity of the pixel is high in a densely connected region that surrounds a large non-contractible cycle of the simplicial complex. The cycle is generated by images that belong to the sequence of digits `7`, `3`, `1`, `9`. Images associated to several nodes along the cycle are shown for reference, with the pixel highlighted in red.

The statistical significance of the combinatorial Laplacian score can be estimated by randomization. For each point cloud feature, it is possible to build a null-distribution for $R_{r}^{\left(q\right)}$ by randomly permuting the labels and computing $R_{r}^{\left(q\right)}$ multiple times. By controlling the rate of type I errors using the standard procedures [17, 18], it is then possible to fix the Čech complex parameter *ε* (or the corresponding parameters of UMAP and Mapper) such that the number of rejected null hypotheses at a fixed false discovery rate (FDR) is maximized. The process is illustrated in Fig 5, where we use the combinatorial Laplacian score to identify informative pixels in the MNIST dataset.

The combinatorial Laplacian score can also be naturally extended to pairs of features, in analogy to the notion of covariance for pairs of random variables (S1 Note). The bivariate combinatorial Laplacian score $R_{r,s}^{\left(q\right)}$ ranks pairs of features according to their adjacency in a simplicial complex. In particular, $R_{r,s}^{\left(q\right)}$ takes low values for pairs of features that take high values in non-overlapping but adjacent regions of the simplicial complex. The bivariate combinatorial Laplacian score thus enables the study of relations between pairs of features based on the topology of the simplicial complex.

We implemented the computation of the 0- and 1-dimensional combinatorial Laplacian score and the 0-dimensional bivariate score in an open-source R package (Methods). In this implementation, the computation of the 0-dimensional combinatorial Laplacian score can be performed in a standard desktop computer for point clouds involving thousands of samples (Table 1). The computation of the 1-dimensional combinatorial Laplacian score is more demanding (Table 1), and usually requires using a high-performance computing environment or subsampling the point cloud.

**Table 1:** Running times of the 0- and 1-dimensional combinatorial Laplacian scores for several simplicial complexes of different size on a standard 8-core desktop computer.

	Vertices	Edges	2-Simplexes	Time
$R_{r}^{\left(0\right)}$	1,000	249,500	-	12m 43s
	1,500	561,750	-	59m 18s
	2,000	999,000	-	3hr 13m 46s
$R_{r}^{\left(1\right)}$	100	600	34,045	41m 14s
	150	2,450	112,175	7hr 19m 6s
	200	5,550	268,758	31hr 48m 28s

### Differential expression analysis

A standard task in genomics is the identification of differentially expressed genes among discrete groups of samples (e.g. tissue specimens or individual cells) whose transcriptome has been profiled using expression microarrays or RNA-sequencing. However, in many situations samples cannot be naturally arranged or clustered into discrete groups based on their transcriptome. Cells (and the tissues they form) often respond to stimuli from their cellular environment in a continuous way. For example, immune cells acquire immunosuppressive or pro-inflammatory phenotypes in different contexts and there is a continuum of cellular phenotypes interpolating among these states. Furthermore, the transcriptome of a cell is the result of many concurrent molecular pathways taking place in the cell. In this section, we utilize the combinatorial Laplacian score to identify differentially expressed genes without predefining groups of samples or cells, as opposed to conventional approaches to differential expression analysis where at least two groups of samples need to be specified. We focus on the particular case of single-cell RNA-seq data.

**Fig 6:**
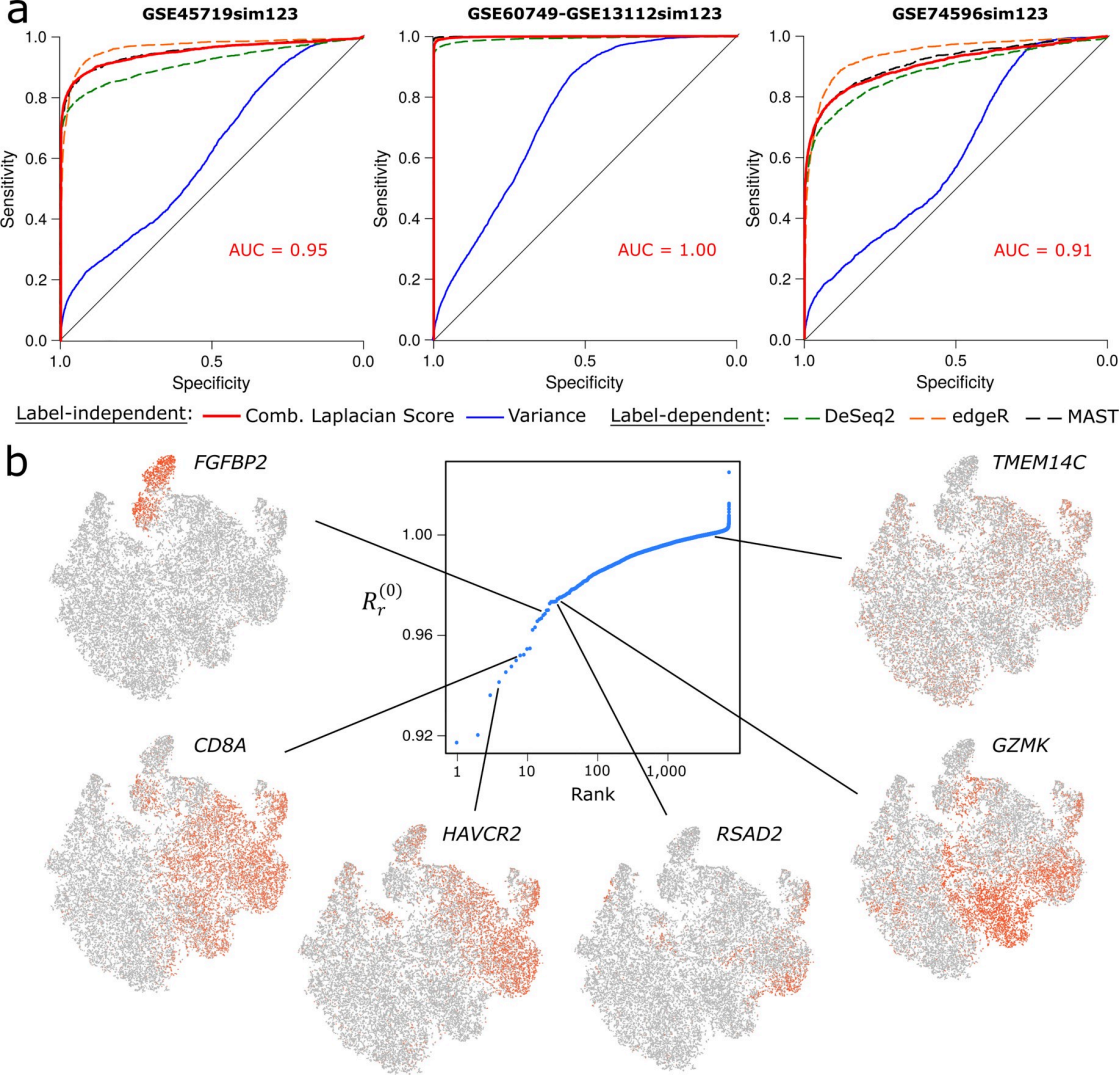
Differential expression analysis using the combinatorial Laplacian score. (a) ROC curves for three simulated scRNA-seq datasets with 10% of the genes being differentially expressed between two populations of cells. The 0-dimensional combinatorial Laplacian score, DeSeq2, edgeR, MAST, and variance were used to identify differentially expressed genes. Conventional methods (DeSeq2, edgeR, and MAST) in addition took as input a list of labels assigning each cell to the corresponding population. (b) Analysis of scRNA-seq data of 24,911 T-cells infiltrating lung tumors and adjacent tissues. Genes were ranked according to their 0-dimensional combinatorial Laplacian score. t-SNE plots color-coded for expression (grey to red) are shown for some of the top differentially expressed genes identified by this method. For reference, the expression of a gene with high combinatorial Laplacian score (TMEM14C) is also displayed. To facilitate interpretation, we use the same t-SNE embedding as in [23].

We assessed the power of the 0-dimensional combinatorial Laplacian score to discriminate differentially expressed genes as compared to conventional methods. To that end, we followed the same approach as in a recent comparative study of single-cell differential expression analysis [19]. We considered three simulated single-cell RNA-seq data sets containing two populations of cells with 10% of the genes being differentially expressed between the populations. These data sets were generated based on three real single-cell RNA-seq data sets (Methods). In our comparisons, we considered three conventional methods for differential expression analysis (DeSeq2 [20], edgeR [21], and MAST [22]), one of which (MAST) is specifically designed for single-cell RNA-seq data. As opposed to the combinatorial Laplacian score, these methods require assigning each cell to a cluster of cells or cellular population and are therefore restricted to situations where such an assignment is possible and stable. Additionally, we also considered variance as an indicator of differentially expressed genes in absence of cell-population or cluster assignments. In all cases, the discriminating power of the combinatorial Laplacian score, as measured by the area under the receiver-operating characteristic curve (AUC), was comparable to that of conventional methods, despite its broader applicability, and was well superior to that of variance (Fig 6A).

We next applied the 0-dimensional combinatorial Laplacian score in a case where conventional methods are of limited utility. Specifically, we considered single-cell RNA-seq data of 24,911 T-cells infiltrating lung tumors and adjacent normal tissue [23]. T-cells in this example do not form well-defined stable clusters according to their transcriptional profile, as evidenced by tSNE representation of the data (Fig 6B). Conventional methods for differential expression analysis do not take into account the instability of clusters and can miss expression patterns that are incompatible with the cluster structure. Ranking genes according to their 0-dimensional combinatorial Laplacian score allowed us to identify differentially expressed genes in this example without having to cluster cells (Fig 6B). Among the top ranked genes are genes whose expression pattern is well accounted for by the cluster structure of the original analysis [23], such as *FGFBP2*, which is expressed exclusively and ubiquitously by natural killer cells. However, this cluster structure does not account for the expression pattern of many of the top differentially expressed genes identified by the combinatorial Laplacian score. For instance, among the cytotoxic CD8+ T-cells, we observe distinct subpopulations of cells expressing combinations of Hepatitis A Virus Cellular Receptor 2 (*HAVCR2*), Radical S-Adenosyl Methionine Domain Containing 2 (*RSAD2*), and Granzyme K (*GZMK*). The combinatorial Laplacian score thus reveals more complex and richer patterns of cellular heterogeneity than clustering-based methods. We expect this approach to be of great utility in studying the transcriptional programs underlying dynamic cellular systems such as those that occur during development, inflammation, or tissue repair [11].

### Cyclic analysis

The application to differential expression analysis described in the previous section only makes use of the graph structure of the simplicial complex. Higher-dimensional combinatorial Laplacian scores can be used to disaggregate differentially expressed genes according to their degree of localization along topological features of the data. For example, in dynamic cellular systems involving convergent lineages, strong cell-cycle effects, or complex differentiation paths, cells may span loops in the expression space. In those situations, the 1-dimensional combinatorial Laplacian score permits identifying genes that are differentially expressed along the loops.

**Fig 7:**
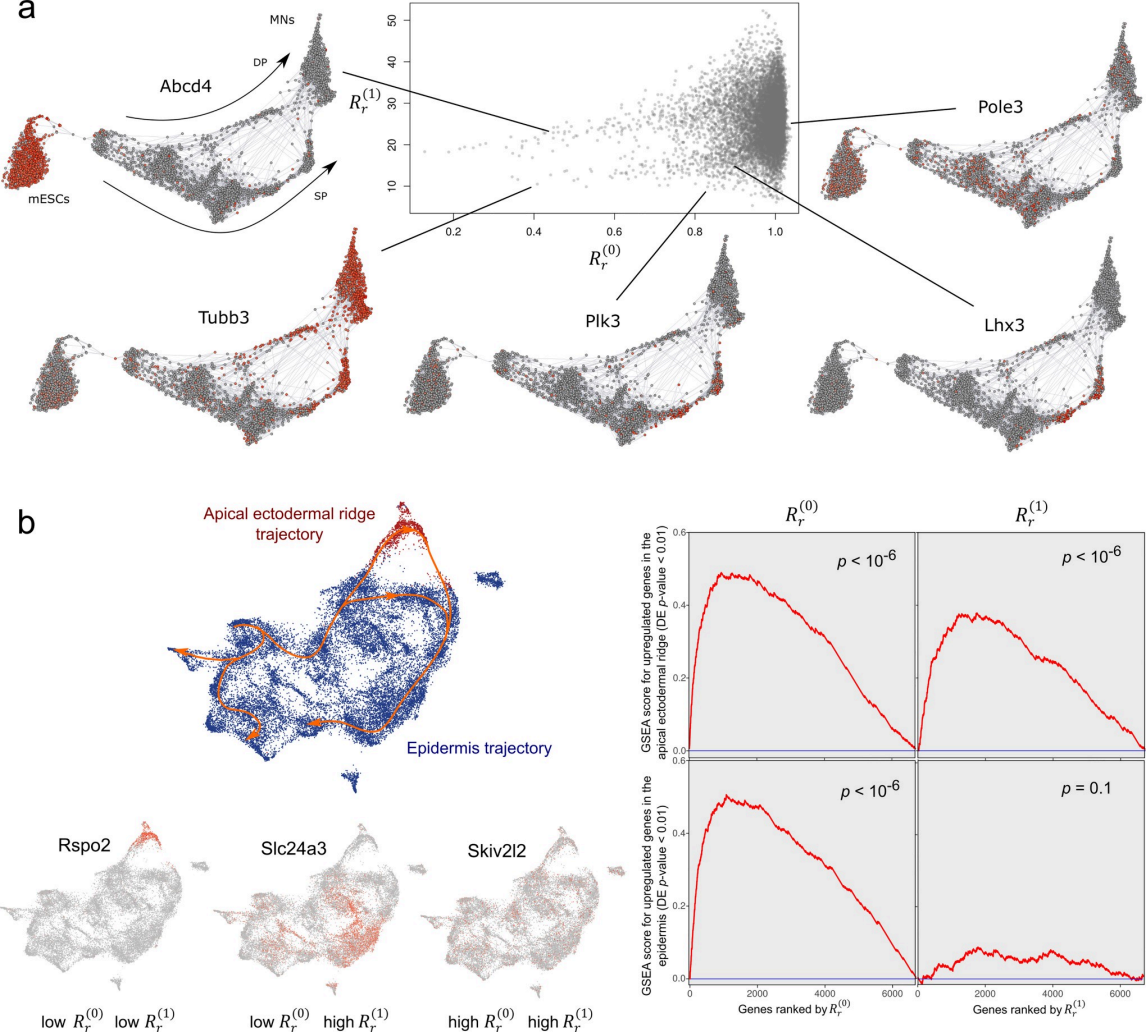
Differential expression analysis of alternative paths of differentiation using the 1-dimensional combinatorial Laplacian score. (a) The 0- and 1-dimensional combinatorial Laplacian scores were run over the scRNA-seq expression data of 3,582 cells from the differentiation of mESCs into MNs using the standard (SP) and direct programming (DP) protocols. The scatter plot represents the 0- and 1-dimensional combinatorial Laplacian scores of 10,063 genes. A k-nearest neighbor graph (k = 4) color-coded for expression (grey to red) is shown for some of the top differentially expressed genes identified by this method. Genes with low values of $R_{r}^{\left(1\right)}$ have upregulated expression along the cycle spanned by the two alternative paths of differentiation. For reference, the expression of a gene with high 0- and 1- combinatorial Laplacian scores (Pole3) is also displayed. (b) The 0- and 1-dimensional combinatorial Laplacian scores were run over the scRNA-seq expression data of 24,930 epithelial cells from the developing mouse (development stages E9.5-E13.5). Apical ectodermal ridge cells form a transient state that branches out and in from epithelial cells. The expression of genes with low value of $R_{r}^{\left(1\right)}$ localizes along the cycle spanned by the two alternative paths of differentiation. Examples of the expression of genes with low 0- and 1-dimensinoal combinatorial Laplacian score, low 0- and high 1-dimensional combinatorial Laplacian score, and high 0- and 1-combinatorial Laplacian score are shown (left).Gene set enrichment analysis (right) shows that genes with low 1-dimensional combinatorial Laplacian score are strongly enriched for genes that are upregulated in the alternative path for differentiation spanned by apical ectodermal ridge cells.

To show the utility of the 1-dimensional combinatorial Laplacian score for differential expression analysis, and cyclic analysis in general, we considered single-cell RNA-seq data of the *in vitro* differentiation of mouse embryonic stem cells into motor neurons using two different protocols [24]. In these experiments, the starting and final transcriptional states are the same between the two differentiation protocols. However, the intermediate transcriptional states are different in each protocol, leading to branching trajectories in the expression space that subsequently converge into the same final state. We ranked the genes in this example according to their 0- and 1-dimensional combinatorial Laplacian scores on a Vietoris-Rips complex constructed from the top 50 principal components of the data (Fig 7A). Genes having a small value of the 1-dimensional combinatorial Laplacian score were differentially expressed at the intermediate states, where the two alternative paths of differentiation occur (S1 Fig, gene-set enrichment analysis (GSEA) *p*-value < 10^−6^). These included genes, such as *Lhx3* and *Plk3*, which are known to be upregulated in one of the two differentiation protocols (Fig 7A). On the other hand, genes having a small value of the 0-dimensional combinatorial Laplacian score but not of the 1-dimensional combinatorial Laplacian score were differentially expressed in the initial or final states of the differentiation process (Fig 7A).

Convergent differentiation lineages also occur *in vivo*. Using highly-parallelized scRNA-seq, Cao et al. [25] recently showed the existence of multiple convergent trajectories in mouse organogenesis which give rise to loops in the expression space. We used the combinatorial Laplacian score to analyze scRNA-seq data of the developing mouse epidermis generated in this study. The apical ectodermal ridge (AER) is a highly specialized transient epithelium involved in digit development [26]. During limb development, the transcriptome of cells from the AER diverges from that of the ectodermal cells to converge at latter stages into the transcriptome of epidermal cells, forming a loop in the expression space (Fig 7B) [25]. We ranked genes in this dataset according to their 0- and 1- dimensional combinatorial Laplacian scores on a Vietoris-Rips complex created from a UMAP representation of the data. Genes with significantly upregulated expression along the AER trajectory not only had a low 0-dimensional combinatorial Laplacian score but also a low 1-dimensional combinatorial Laplacian score (Fig 7B and S2 Fig, GSEA p-value < 10^−6^). On the other hand, genes with significantly upregulated expression in other regions of the expression space only had a low 0-dimensional combinatorial Laplacian score (Fig 7B and S2 Fig, GSEA p-value < 10^−6^) but not a low 1-dimensional score (Fig 7B and S2 Fig, GSEA p-value = 0.08). Thus, the 1-dimensional combinatorial Laplacian score is able to disaggregate differentially expressed genes according to their association to alternative paths of differentiation without making use of any labels.

Loops in the expression space of cells can also be produced by other effects unrelated to the convergence of differentiation trajectories, such as cell cycle effects. In a previous study, the Mapper algorithm was used to construct a topological representation of the expression space of the *in vitro* differentiation of mouse embryonic stem cells (mESCs) into motor neurons (MNs) [11]. In that work, the presence of multiple loops in the region of neural precursors was hypothesized to be related to cell proliferation. To further explore the potential biological origin of these loops, we revisited this dataset and computed the 0- and 1-dimensional combinatorial Laplacian scores of gene expression on the Mapper simplicial complex (S3 Fig). Consistent with our expectations, the expression of genes that have a low 1-dimensional combinatorial Laplacian score appeared localized around the non-contractible loops of the representation (S3 Fig). Gene ontology enrichment analysis revealed regulation of cell proliferation (GO:0042127, GSEA *q*-value = 2×10^−7^) as the most enriched biological process among genes in the lowest quartile of both the 1-dimensional and the 0-dimensional Laplacian scores. To the contrary, the most enriched ontologies among genes in the lowest quartile of the 0-dimensional Laplacian score but the top quartile of the 1-dimensional Laplacian score were neuron differentiation (GO:0030182, GSEA *q*-value = 5×10^−10^), axonogenesis (GO:0007409, GSEA *q*-value = 5×10^−9^), and other non-cyclic biological processes.

Taken together, these three examples show that, when the expression space has a complex topology, different combinatorial Laplacian scores allow the identification and categorization of differentially expressed genes according to the topology of the expression space without pre-defining cellular populations or clustering cells.

### Multi-modal data analysis

Spectral methods rank features according to their degree of consistency with a given metric structure, as we have described. However, the data type of the features may differ from that of the metric structure. Spectral methods can therefore be adapted to perform multi-modal analyses, where features of a given type are ranked according to their degree of consistency with a metric structure of a different type. To show the utility of this type of analysis, we considered two specific applications to multi-modal genomic data.

**Fig 8:**
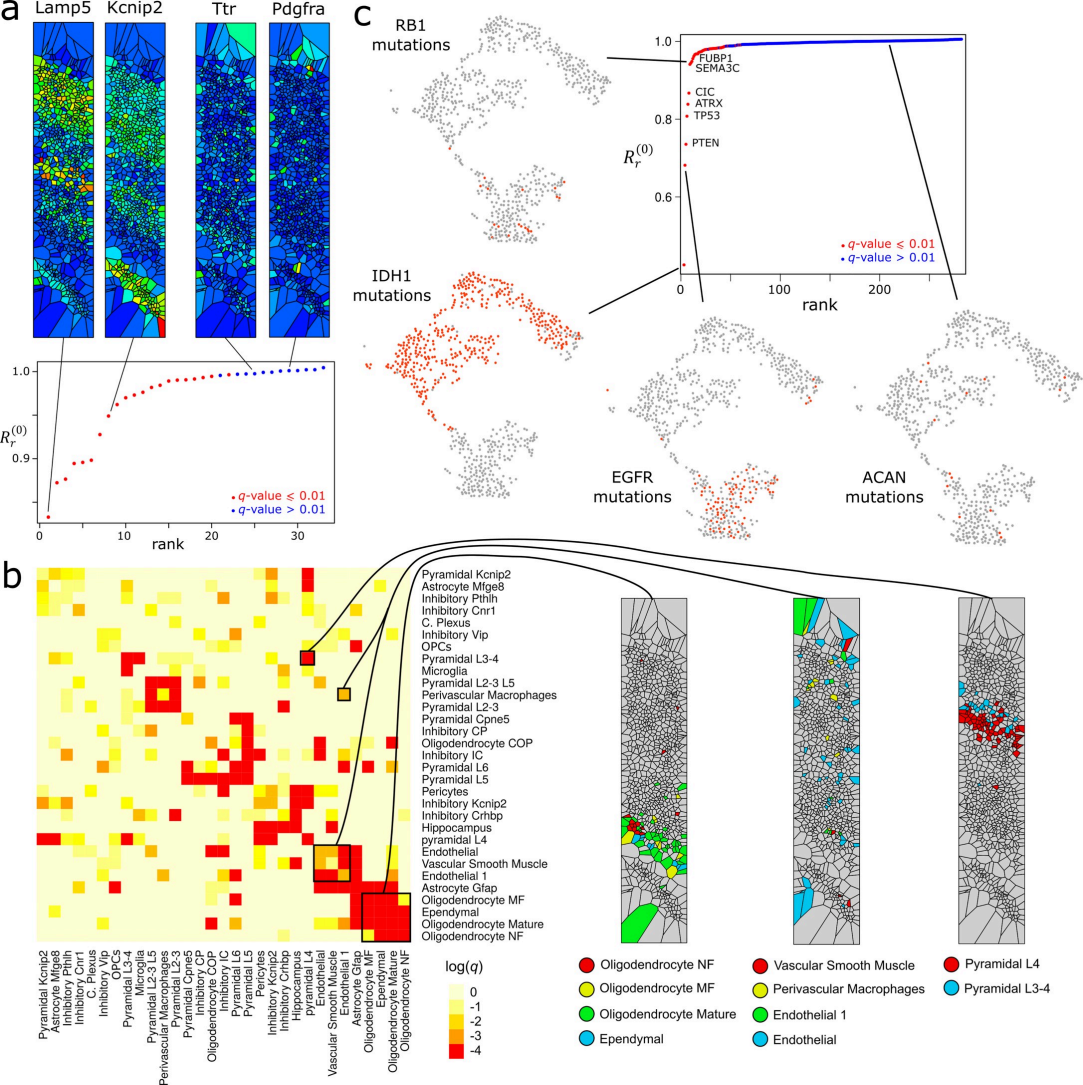
Analysis of multi-modal genomic data using the univariate and bivariate combinatorial Laplacian scores. (a) The 0-dimensional Laplacian score was utilized to identify genes that are differentially expressed across spatial directions in a section of the murine somatosensory cortex using single-molecule FISH data. Gene expression levels were evaluated using the combinatorial Laplacian score based on the spatial dimensions. Genes with low $R_{r}^{\left(0\right)}$ have significant spatial patterns of expression. For reference, the expression patterns of two genes with low $R_{r}^{\left(0\right)}$ (Lamp5 and Kcnip2) and two genes with high $R_{r}^{\left(0\right)}$ (Ttr and Pdgfra) are shown. Cells are represented by means of a Voronoi tessellation and are color-coded according to the expression level of the gene (blue: low; red: high). (b) Analysis of spatial relations among cell populations using the bivariate combinatorial Laplacian score. The 0-dimensional bivariate combinatorial Laplacian score was computed for each pair of cell populations in the murine somatosensory cortex dataset using the spatial dimensions to build the Vietoris-Rips simplicial complex. The significance of the score was estimated for each pair of cell populations by randomization and is shown in the heatmap. Cells from pairs of populations that have a significant score often appear adjacent to each other in the spatial dimensions. (c) Analysis of whole exome and mRNA sequencing data of 667 low-grade glioma and glioblastoma tumors of The Cancer Genome Atlas (TCGA) using the 0-dimensional Laplacian score. A Vietoris-Rips complex was built based on the gene expression data. Binary vectors indicating whether a gene is non-synonymously mutated or not were taken as features. The 0-dimensional Laplacian score identifies genes for which their mutation is associated with consistent global expression patterns. A UMAP representation based on the expression data is shown for some representative genes with low (IDH1, EGFR, and RB1) or high (ACAN) value of $R_{r}^{\left(0\right)}$, color-coded according to the somatic mutation status of the gene (orange: non-synonymously mutated; grey: non-mutated or synonymously mutated).

Using spatially-resolved transcriptomics, the expression level of genes can be measured in their spatial context, often with single-cell resolution. We considered single-molecule fluorescence *in-situ* hybridization (FISH) data of the murine somatosensory cortex [27] and utilized the combinatorial Laplacian score to rank and disaggregate genes according to their spatial expression pattern (Fig 8A). Genes with low values of the 0-dimensional Laplacian score were spatially patterned, being often expressed in only a subset of the cortical layers. To identify spatial relations among distinct cell populations, we applied the 0-dimensional bivariate combinatorial Laplacian score to the published cell assignments in this data (Fig 8B). This analysis identified pairs of cell populations that are located adjacent to each other in the FISH slide. Such pairs often consisted of neuronal populations residing at consecutive cortical layers or cell constituents of the same tissue structure, such as blood vessels (Fig 8B).

Another instance where multi-modal data analysis is of prime importance is quantitative trait association studies. These studies seek to establish associations between quantitative phenotypes (e.g. gene expression levels) and genotypes. To demonstrate the utility of the combinatorial Laplacian score to establish associations between genetic variants and complex high-dimensional phenotypic spaces, we analyzed somatic mutation data, obtained by whole-exome sequencing, and mRNA expression data of a cohort of 667 low-grade glioma and glioblastoma tumors [28]. We used the 0-dimensional combinatorial Laplacian score to identify non-synonymous mutations that are associated with consistent global expression patterns (Fig 8B). Among the mutated genes with significant 0-dimensional Laplacian score (Benjamini-Hochberg adjusted *q*-value ≤ 0.01; *n* = 1,000 permutations), we observed numerous known cancer-associated genes in adult brain glioma. The significance of this observation was confirmed by a gene-set enrichment analysis using the recurrently-mutated genes reported by MutSig2CV in the same cohort (GSEA *p*-value < 10^−4^). These results demonstrate the potential of the Laplacian score to establish associations among multiple types of genomic data when samples cannot be arranged into discrete classes.

### Summary and discussion

Modern data often comes in the form of large point clouds. A common approach to analyzing these point clouds involves clustering the data based on the underlying metric structure and using some statistical test to identify features that differ across clusters. However, there are multiple situations where data cannot be naturally structured into clusters. Even in the cases where clustering is possible, there are always basic trade-offs inherent to the clustering problem [29]. Moreover, in this two-step approach the uncertainty in the clustering step is usually not taken into account in downstream statistical tests, giving rise to potentially misleading conclusions. Spectral methods bypass the clustering step and directly use the underlying metric structure of the data to perform feature selection. However, current spectral methods are graph-based and limited to point features of the data. In this work we have extended these methods and presented a general framework for feature selection based on simplicial complexes built from the data, establishing a connection between existing manifold learning and topological data analysis approaches. This generalization allows us to take into account the topological structure associated with the data as well as to perform unsupervised feature selection on features that have a complex combinatorial structure (for instance, defined over pairs of points, triplets, etc.). Apart from extending the Laplacian score to higher-order combinatorial structures, our work introduces several other novel aspects in the original formulation of the Laplacian score. Specifically, we propose a statistical framework for the score based on randomization which allows us to fix the parameters of feature selection by maximizing the statistical power; we show the utility of the score to establish multi-modal associations by considering different data modalities for the metric and the features; and we generalize the Laplacian score to study relations between pairs of features. In the context of gene expression, our approach supplements recently-developed algorithms for the analysis of expression patterns [30, 31].

The framework we have introduced has natural applications in the analysis of genomic data when the data cannot be comprehensively and stably described by clusters. This situation occurs frequently in datasets consisting of many samples, such as those derived from high-throughput single-cell omics experiments (thousands of cells) or large cross-sectional studies (thousands of specimens). The resolution of these datasets is such that they often capture continuous patterns of variation that cannot be faithfully represented by clusters. Some of these studies involve multiple concurrent data modalities (e.g. transcriptomic, proteomic, epigenetic, genomic, phenotypic, etc.) and we have shown that spectral simplicial methods can be also naturally applied in those situations. As the scale and complexity of genomic datasets continue to increase, we expect these methods will become a particularly useful tool for the analysis of genomic data.

## Methods

### Software implementation

The 0- and 1- dimensional combinatorial Laplacian scores are implemented in the open-source R package RayleighSelection (https://github.com/CamaraLab/RayleighSelection). To improve performance, we used parallelization and embedded C++ code for the computationally demanding parts. Additionally, we included routines for the generation and visualization of simplicial complexes.

### Analysis of MNIST data set

We performed feature selection on the MNIST dataset using the 0- and 1-dimensional Laplacian scores. Each sample consists of a 28x28 grey-scale image of a handwritten digit. In our analysis, each feature represents the intensity of a pixel across all images. A Vietoris-Rips complex was built from the Pearson’s correlation distance between 100 handwriting samples using the top 25% pixels with highest variance. The Vietoris-Rips parameter was set to *ε* = 0.7, as determined by maximization of the number of rejected null hypotheses at a fixed FDR of 0.05 (Benjamini-Hochberg procedure). The *p*-values were estimated by randomization with 5,000 permutations.

### Comparisons using simulated single-cell expression data

We used the 0-dimensional Laplacian score to identify differentially expressed genes in three simulated datasets generated by Soneson and Robinson [19] based on real single-cell expression data (GEO Database accession numbers GSE45719, GSE74596, GSE60749-GPL13112). These simulated datasets contain two populations of 50 cells such that 10% of the genes are differentially expressed between the two populations. A Vietoris-Rips complex was created from Pearson’s correlation distance using the top 25% genes with highest variance, and the value of *ε* was in all cases determined by maximizing the number of rejected null hypothesis at FDR = 0.05. The *p*-value of the 0-dimensional Laplacian score was calculated for each gene by randomizing the data with 5,000 permutations. We then computed the AUC by comparing with the actual simulated differentially expressed genes. We ran the software edgeR [21] using the quasi-likelihood general linear model (GLM), MAST, and DESeq2 with default parameters on the same dataset, providing them with the population identifier of each cell.

### Analysis of T-cells infiltrating lung tumors and adjacent tissue

We performed differential expression analysis of single-cell RNA-seq data from 24,911 T-cells infiltrating lung tumors and adjacent normal tissue from Lambrechts et al. [23]. We used Pearson’s correlation distance to build a Vietoris-Rips complex and determined the parameter of the complex by maximizing the number of rejected null hypothesis (*ε* = 0.7, FDR = 0.1). To reduce the computational time, we only used 1,000 randomly sampled cells and the top 2,800 genes with highest variance to build the simplicial complex. Expression values were transformed to log(1+TPM). We computed the 0-dimensional Laplacian score for the 7,242 genes that were expressed in more than 1% and less than 25% of cells.

### Analysis of alternative paths of motor neuron differentiation

To demonstrate the efficacy of the 1-dimensional Laplacian score in the analysis of genomics data, we considered the combined scRNA-seq data of two differentiation protocols on mouse embryonic stem cells (GEO Database accession number GSE97390) from Briggs et al. [24]. We processed the data in the same way as in the original publication. In brief, we used the force-directed visualization package SPRING [32] with the same parameters as described in the quick start guide (https://github.com/AllonKleinLab/SPRING) to visualize and confirm the cyclic nature of the expression data. The first 50 principal components output by SPRING were then used to calculate a Euclidean distance matrix and a Vietoris-Rips complex for 200 randomly sampled cells, where the value of the parameter was determined by maximizing the number of rejected null hypothesis (*ε* = 28, FDR = 0.1). An orientation in the complex was determined by ordering the cells according to their graph distance from one of the terminally differentiated cells in the SPRING representation. We computed the 0-dimensional and 1-dimensional Laplacian scores for all the genes with expression detected in more than 5% and less than 50% of the cells. Statistical significance was estimated by randomization with 5,000 permutations. Using edgeR’s GLM fold-change threshold test (log fold change > 1), we performed differential expression analysis between the intermediate state cells, as defined in Briggs et al. [24], and all other cells in the combined dataset. We used GSEA to determine the enrichment of significantly differentially expressed genes (p-value < 0.01) based on the ordering of the 0- and 1-dimensional Laplacian scores. The significance of the GSEA score was calculated from a null distribution built using one million permutations of the gene labels.

### Analysis of murine epidermal developmental trajectory

We extracted cells of the epidermis and AER trajectories from the scRNA-seq dataset published by Cao et al. [25] and applied Seurat 3 [33] to preprocess and visualize the trajectories. A UMAP representation was built based on the top 30 principal components, using cosine distance metric and parameters *min*.*dist* = 0.1, *n*.*neighbors* = 15. We used the 0-dimensional and 1-dimensional Laplacian scores to rank gene expression on a Vietoris-Rips complex created from this UMAP embedding (*ϵ* = 2.4) for all genes expressed in more than 2% and less than 50% of cells. Orientation in the simplicial complex was determined by ordering cells based on their Euclidean distance from a chosen cell at the earliest timepoint. We performed differential expression analysis between the two trajectories annotated by Cao et al. [25] using edgeR. We then did GSEA to assess whether differentially expressed genes (edgeR GLM log fold change > 1, p-value < 0.01) tend to have high or low 0-dimensional and 1-dimensional Laplacian scores. Significance of this enrichment was computed using a null distribution based on one million permutations of the gene labels.

### Analysis of cell cycle effects in motor neuron differentiation

We used the combinatorial Laplacian score to reanalyze the Mapper representation of a motor neuron differentiation dataset previously generated in Rizvi et al. [11]. Mapper builds a simplicial complex over a point cloud by first defining a covering of the point cloud based on a low-dimensional representation. Within each patch in the cover, points are clustered using single linkage hierarchical clustering. A simplicial complex is then formed such that each node corresponds to a cluster of points (cells in this particular example), edges correspond to non-zero pairwise intersections between clusters, triangles correspond to non-zero triple intersections among clusters, etc. Using this simplicial complex, we computed the 0-dimensional and 1-dimensional Laplacian score of each gene with expression detected in more than 10% and less than 50% of the cells. The orientation of the simplicial complex was specified based on the timepoints the cells were sampled. The expression value of a gene at each node in the simplicial complex was calculated as the average of its expression level over all cells in that node. We used the algorithm g:Profiler [34] to perform gene ontology enrichment analysis, using as a background the full list of genes for which we computed the Laplacian score. We performed gene ontology enrichment analysis on all genes in the lowest quartile of the 1-dimensional Laplacian score and the lowest quartile of the 0-dimensional Laplacian score, ordered by their 1-dimensional Laplacian score. For comparison, we also performed gene ontology enrichment analysis on genes in the top quartile of the 1-dimensional Laplacian score and the lowest quartile of the 0-dimensional Laplacian score, in reverse order by their 1-dimensional Laplacian score.

### Analysis of murine somatosensory cortex data

We considered single-molecule FISH data generated by Codeluppi et al. [27] for a 325 x 600 μm region of the murine somatosensory cortex. We filtered out any cells that were less than 4 μm^2^ or greater than 338 μm^2^ in size or contained fewer than 40 total molecules. We then used Euclidean distance between the spatial coordinates of the remaining cells to create a Vietoris-Rips complex (*ε* = 6,500). We computed the 0-dimensional Laplacian score of the expression of each gene normalized by the total number of cell molecules. Using the same cells, we created a Vietoris-Rips complex (*ε* = 1,000) to compute the bivariate combinatorial Laplacian score of the binary cell type assignments. Significance of the bivariate score was computed based on 2,000 random permutations of the cell type labels. We visualized the expression of specific genes and cell populations using a Voronoi tessellation centered on the spatial coordinates of the cells.

### Analysis of expression and mutation data of adult gliomas

We re-analyzed somatic mutation data and mRNA expression data of a cohort of 667 low-grade glioma and glioblastoma tumors from TCGA [28]. We created a Vietoris-Rips complex (*ε* = 0.22) using Pearson’s correlation distance and the highest-variance genes (σ > 1) in the log-transformed expression data. Binary vectors indicating the presence of at least one non-synonymous somatic mutation in each gene were taken as features, and the 0-dimensional Laplacian score was computed on these features. To verify that the genes with significant Laplacian scores (q-value ≤ 0.01, 1,000 permutations) are cancer-associated, we performed a gene set enrichment analysis (null distribution built using 10,000 permutations) of the genes reported by MutSig2CV [35] in the list of genes ranked by their Laplacian score. Specific genes were visualized on a UMAP [14] representation of the expression data.

## Supporting information

S1 Note Spectral simplicial theory for feature selection. Mathematical appendix introducing the definition and properties of the combinatorial Laplacian score on simplicial complexes and its application to feature extraction.
(PDF)

S1 Fig Gene-set enrichment analysis for upregulated genes at intermediate (top) or early/late (bottom) states in the example of the in vitro differentiation of mESCs into MNs using standard and direct programming protocols. Genes are ranked according to their 0- (left) and 1-dimensional (right) combinatorial Laplacian score. Genes with 0-dimensional combinatorial Laplacian score are enriched for genes that are differentially expressed at any stage within the differentiation. Genes with low 1-dimensional combinatorial Laplacian score are strongly enriched for genes that are upregulated at intermediate states, where the alternative paths for differentiation occur.
(TIF)

S2 FigDifferential expression analysis of convergent cell differentiation trajectories in the developing mouse epidermis using the 1-dimensional combinatorial Laplacian score.The scatter plot represents the 0- and 1- dimensional combinatorial Laplacian scores of 6,691 genes. A UMAP representation color-coded for expression (grey to red) is shown for some of the top differentially expressed genes identified by this method. Genes with low values of $R_{r}^{\left(1\right)}$ have upregulated expression along the AER trajectory. For reference, the expression of a gene with high 0- and 1- combinatorial Laplacian scores (*Skiv2l2*) is also displayed.(TIF)

S3 FigDifferential expression analysis of the in vitro differentiation of mESCs into MNs using the 1-dimensional combinatorial Laplacian score.The 0- and 1-dimensional combinatorial Laplacian scores were run over the scRNA-seq expression data of the differentiation of mESCs into MNs using the SP protocol. The scatter plot represents the 0- and 1-dimensional combinatorial Laplacian scores of 6,938 genes. A Mapper simplicial complex color-coded for expression (blue: low expression; red: high expression) is shown for some of the top differentially expressed genes identified by this method. Genes with low values of $R_{r}^{\left(1\right)}$ have upregulated expression along the loops in the region of the neural precursors, where cell cycle effects are large. For reference, the expression of a gene with high 0- and 1- combinatorial Laplacian scores (*Nf1*) is also displayed.(TIF)
