# Supplementary material for: Clustering-independent analysis of genomic data using spectral simplicial theory
Source: PLoS Comput Biol. 2019 Nov 22;15(11):e1007509. doi: 10.1371/journal.pcbi.1007509 (PMC6897424; doi:10.1371/journal.pcbi.1007509)
Supplement: S1 Note — Mathematical appendix introducing the definition and properties of the combinatorial Laplacian score on simplicial complexes and its application to feature extraction. (PDF) [file pcbi.1007509.s001.pdf]

SUPPLEMENTARY NOTE:

Spectral Simplicial Theory for Feature Selection

Kiya W. Govek*, Venkata S. Yamajala*, and Pablo G. Camara#

Department of Genetics and Institute for Biomedical Informatics,
Perelman School of Medicine, University of Pennsylvania,
3400 Civic Center Blvd., Philadelphia, PA 19104.

* These authors contributed equally to this work.

# Correspondence to: pcamara@pennmedicine.upenn.edu

The main objects of study in this paper are a finite data set (often also termed as point cloud) X with a notion of distance or dissimilarity $\|x_i - x_j\|$, where $x_i, x_j \in X$ and $\|\cdot\|$ is a distance in X, and a set of features $\{f_r\}$ defined as maps from X into a formally real field F. In 2006, He, Cai, and Niyogi proposed an algorithm for unsupervised feature selection called Laplacian score (1). They construct a weighted nearest neighbor graph G with nodes X and adjacency matrix A and introduce an inner product in G defined by

$$\langle f_r, f_s \rangle_G = \sum_{x_i \in X} w(x_i)\, f_r(x_i)\, f_s(x_i)$$

with $w(x_i) = \sum_j A_{ij}$. The Laplacian score of the r-th feature is then given by the Rayleigh quotient of the normalized graph Laplacian with respect to this inner product,

$$R_r = \frac{\langle \tilde{f}_r, L_G \tilde{f}_r \rangle_G}{\langle \tilde{f}_r, \tilde{f}_r \rangle_G}$$

where

$$L_G = I - \mathrm{diag}(w)^{-1} A$$

$$\tilde{f}_r = f_r - \frac{\langle f_r, \mathbf{1} \rangle_G}{\langle \mathbf{1}, \mathbf{1} \rangle_G}$$

and $\mathbf{1}(x_i) = 1, \forall x_i \in X$, is the unit feature vector. The Laplacian score ranks features according to their consistency with the structure of G. Specifically, features with small values for R_r take high values in highly connected nodes of G. This approach to unsupervised feature selection has become widespread, as it offers a substantial statistical power compared to ranking features according to their variance (1). In what follows, we generalize these notions to simplicial complex representations of the data.

Preliminary Definitions. We first recall some standard definitions from algebraic topology that will be used below (2). We define an ordered abstract simplicial complex K on a finite set $V = \{v_0, \ldots, v_M\}$ as a collection of ordered subsets of V which is closed under inclusion, i.e. $\tau \subset \sigma \Rightarrow \tau \in K,\ \forall \sigma \in K$. The $(q+1)$-dimensional elements of K are called q-simplices. We denote the set of q-simplices of K by $S_q(K)$.

There are multiple ways to construct an ordered abstract simplicial complex from a data set X and an order relation $x_0 < x_1 < \cdots < x_N$ among the elements of X (3). The Čech complex $\mathrm{C}(X, \varepsilon)$ is defined as the abstract simplicial complex that results from considering the set of open balls with radius $\varepsilon \in \mathrm{F}^+$ centered at the elements of X, where balls correspond to 0-simplices, pairwise intersections to 1-simplices, triple intersections to 2-simplices, etc. The utility of the Čech complex is ensured by the nerve theorem: if the elements of X were sampled from a topological space $\mathcal{M}$, under certain sampling assumptions the Čech complex approximates the topology of $\mathcal{M}$. In particular, the 1-skeleton (i.e. the set of 0- and 1-simplices) of the Čech complex is a graph of the type considered in manifold learning approaches. Since computing triple and higher-order intersections among balls is computationally costly, the Vietoris-Rips complex (defined as the clique complex of the 1-skeleton of a Čech complex) is often used in practice as an approximation to the Čech complex (3). Specifically, given a Čech complex, there

exists a chain of inclusion maps among the Čech complex and two Vietoris-Rips complexes such that the Čech complex is in the middle (4).

Given a $(q+1)$-simplex $\sigma = \{v_0, \dots, v_{q+1}\} \in K$, we define its boundary as the linear combination of q-simplices

$$\partial\sigma = \sum_{k=0}^{q+1} (-1)^k \{v_0, \dots, v_{k-1}, v_{k+1}, \dots, v_{q+1}\}$$

where 1 is the unit element of F. More generally, the boundary operator can act on linear combinations of q-simplices. We denote by $\mathrm{sgn}(\tau, \partial\sigma)$ the sign of a q-simplex τ contained in the boundary of σ.

We now turn our attention to maps from X into F. We define a q-point feature of X as a map $f\left(x_{i_1}, \dots, x_{i_q}\right)$ from X^q into F. For instance, if the elements of X are trading agents, the frequency two agents enter into a commercial transaction to trade a specific type of asset would constitute a 2-point feature of X. Similarly, in a metabolic network with vertices representing metabolites and edges representing metabolic reactions catalyzed by enzymes, enzyme levels would be represented as a 2-point feature. Every q-point feature of X induces a map from the $(q-1)$-simplices of the Čech complex $\mathrm{C}(X, \varepsilon)$ into F in the obvious way. More generally, we define a discrete q-form or q-cochain $f^{(q)}$ on a simplicial complex as a map from $S_q \subset K$ into F. If the simplicial complex is obtained by discretizing a manifold, for instance through triangulation, discrete q-forms on K approximate differential q-forms on the manifold (5). In the case of reduced abstract simplicial complex representations, such as those produced by Mapper, 1-point features can also induce q-forms with $q > 1$.

With these definitions we can extend the inner product $\langle\, , \rangle_G$ to a weighted inner product between q-forms on simplicial complexes

$$\langle f_r^{(q)}, f_s^{(q)} \rangle_K = \sum_{\tau \in S_q} w(\tau)\ f_r^{(q)}(\tau)\, f_s^{(q)}(\tau)$$

where $w(\tau) \in \mathrm{F}$ is the weight of τ. In particular, notice the inner product between 0-forms is equivalent to $\langle\, ,\rangle_G$ where G is the 1-skeleton of K.

Finally, we introduce Eckmann's generalization of the graph Laplacian to discrete q-forms on simplicial complexes (6), namely the combinatorial Laplacian $L_K^{(q)}$ on simplicial complexes, defined as

$$L_K^{(q)} = L_K^{(q),\uparrow} + L_K^{(q),\downarrow}$$

$$(L_K^{(q),\uparrow} f_r^{(q)})(\tau) = \sum_{\substack{\sigma \in S_{q+1}: \\ \tau \in \partial\sigma}} \frac{w(\sigma)}{w(\tau)} f_r^{(q)}(\tau) + \sum_{\substack{\tau' \in S_q:\, \tau \neq \tau', \\ \tau, \tau' \in \partial\sigma}} \frac{w(\sigma)}{w(\tau)} \operatorname{sgn}(\tau, \partial\sigma)\, \operatorname{sgn}(\tau', \partial\sigma)\, f_r^{(q)}(\tau')$$

$$(L_K^{(q),\downarrow} f_r^{(q)})(\tau) = \sum_{\rho \in \partial\tau} \frac{w(\tau)}{w(\rho)} f_r^{(q)}(\tau) + \sum_{\substack{\tau': \\ \tau \cap \tau' = \rho}} \frac{w(\tau')}{w(\rho)} \operatorname{sgn}(\rho, \partial\tau)\, \operatorname{sgn}(\rho, \partial\tau')\, f_r^{(q)}(\tau')$$

In these expressions $L_K^{(q),\uparrow}$ only depends on S_{q+1}, whereas $L_K^{(q),\downarrow}$ only depends on S_{q-1}. In particular, $L_K^{(0)} = L_K^{(0),\uparrow}$ is independent of the ordering of X (as $\operatorname{sgn}(\tau, \partial\sigma)\, \operatorname{sgn}(\tau', \partial\sigma) = \operatorname{sgn}(\tau, -\partial\sigma)\, \operatorname{sgn}(\tau', -\partial\sigma) = -1, \forall \sigma \in S_1$, where $\tau, \tau' \in \partial\sigma$ and $\tau \neq \tau'$) and is identical to the graph Laplacian L_G, where G is the 1-skeleton of K. Furthermore, if K is the result of discretizing a manifold, the normalized combinatorial Laplacian approximates the continuous Laplace-Beltrami operator of the manifold (5). With these elements at hand, we are now ready to introduce the combinatorial Laplacian score for q-point features.

Combinatorial Laplacian Score. Given a simplicial complex representation K of a finite data set and a set of features, we define the combinatorial Laplacian score as

$$R_r^{(q)} = \frac{\langle \tilde{f}_r^{(q)}, L_K^{(q)} \tilde{f}_r^{(q)} \rangle_K}{\langle \tilde{f}_r^{(q)}, \tilde{f}_r^{(q)} \rangle_K}$$

where

$$\tilde{f}_r^{(q)} = f_r^{(q)} - \frac{\langle f_r^{(q)}, \mathbf{1}^{(q)} \rangle_K}{\langle \mathbf{1}^{(q)}, \mathbf{1}^{(q)} \rangle_K}$$

In this expression, $\{f_r^{(q)}\}$ are the discrete q-forms on K induced by the set of features, and $\mathbf{1}^{(q)}: S_q \to 1$ denotes the unit q-form on K. Hence, the combinatorial Laplacian score for 0-forms $R_r^{(0)}$ reduces to the ordinary Laplacian score on the 1-skeleton graph of K. For higher-order forms, the combinatorial Laplacian score ranks features according to their degree of localization along homological features of the simplicial complex.

Relation to Feature Extraction. Feature extraction is a closely related problem to feature selection, where a finite set of synthetic features that optimally capture the structure of the point cloud is engineered. These synthetic features can be then used for dimensionality reduction and de-noising. The Laplacian score of He, Cai, and Niyogi follows from the Laplacian Eigenmaps for dimensionality reduction (7). In what follows, we show how this relation can be naturally extended to simplicial complexes and combinatorial Laplacian Eigenmaps.

To that end, we consider the diagonalization problem of the combinatorial Laplacian,

$$\left(L_K^{(q)} y_i^{(q)}\right)(\tau) = \lambda_i^{(q)} y_i^{(q)}(\tau)$$

Using the Courant-Fischer-Weyl min-max principle we can express the eigenvalues of the combinatorial Laplacian in terms of a feature optimization problem with respect to the combinatorial Laplacian score,

$$\lambda_i^{(q)} = \max_{\substack{U: \\ \dim(U) = \dim(S_q) - i + 1}} \left\{ \min_{\tilde{f}_r^{(q)} \in U} R_r^{(q)} \right\}$$

From this relation we observe that the eigenvectors of the combinatorial Laplacian minimize the combinatorial Laplacian score successively across maximal orthogonal directions. In particular, the combinatorial Laplacian score is bounded by the smallest and largest eigenvalues of the combinatorial Laplacian, $\lambda_1^{(q)} \leq R_r^{(q)} \leq \lambda_{\dim(S_q)}^{(q)}$.

We define the m-dimensional combinatorial Laplacian Eigenmap for q-simplices as the map from S_q into $\mathbb{R}^m$ given by

$$\begin{aligned} \mathcal{M}^{(q)}\colon S_q &\to \mathbb{R}^m \\ \tau &\mapsto \left(y_1^{(q)}(\tau), \dots, y_m^{(q)}(\tau)\right) \end{aligned}$$

In particular, $\mathcal{M}^{(0)}$ reduces to the ordinary Laplacian Eigenmap in the 1-skeleton of K. The combinatorial Laplacian Eigenmap for q-simplices thus provide locally-preserving low-dimensional representations of the data where each point in the reduced representation corresponds to $q + 1$ related points in X.

Bivariate Combinatorial Laplacian Score. The combinatorial Laplacian score for discrete differential forms can be thought in close analogy to the concept of variance for random variables. In this regard, it is natural to extend the combinatorial Laplacian score to pairs of discrete differential forms, similarly to the covariance of pairs of random variables,

$$R_{r,s}^{(q)} = \frac{\langle \tilde{f}_r^{(q)}, L_K^{(q)} \tilde{f}_s^{(q)} \rangle_K}{\left| \langle \tilde{f}_r^{(q)}, \tilde{f}_s^{(q)} \rangle_K \right|}$$

We call $R_{r,s}^{(q)}$ the bivariate combinatorial Laplacian score of a pair of q-point features. For 0-forms, $\langle \tilde{f}_r^{(0)}, \tilde{f}_s^{(0)} \rangle_K = \mathrm{cov}_w\left(f_r^{(0)}, f_s^{(0)}\right)$ reduces to the weighted covariance of $f_r^{(0)}$ and $f_s^{(0)}$, and

$$\langle \tilde{f}_r^{(0)}, L_K^{(0)} \tilde{f}_s^{(0)} \rangle_K = \langle f_r^{(0)}, L_G f_s^{(0)} \rangle_G$$

$$= \frac{1}{2} \sum_{x_i, x_j \in X} \left(f_r^{(0)}(x_i)\, f_s^{(0)}(x_i) + f_r^{(0)}(x_j)\, f_s^{(0)}(x_j) - 2 f_r^{(0)}(x_i)\, f_s^{(0)}(x_j) \right) A_{ij}$$

where G is the 1-skeleton graph of K. Thus, $R_{r,s}^{(0)}$ is small for pairs of features that take mutually-exclusive high values in adjacent nodes of G (e.g. $f_r^{(0)}(x_1) = 1$, $f_r^{(0)}(x_2) = 0$, $f_s^{(0)}(x_1) = 0$, $f_s^{(0)}(x_2) = 1$, $A_{12} = 1$). Note in particular that when the two forms are identical, $R_{r,s}^{(q)}$ reduces to the combinatorial Laplacian score introduced above.

Directions for Future Research. There are at least two directions that we believe deserve further investigation. Graph Laplacians admit elegant interpretations in terms of random walks, Markov chains, and diffusion processes (8). It is still unclear to us how this formalism can be generalized to higher-dimensional combinatorial Laplacians and random walks on simplicial complexes, although some progress has already been done in that direction (9, 10). Additionally, given a filtration of Čech complexes with varying scale ε, it has been shown that its cohomology (spanned by the zero eigenvalue q-forms of the combinatorial Laplacian) can be formulated in terms of the theory of persistence (11). It is an open question whether this formulation can be extended to non-zero eigenvalues of the combinatorial Laplacian. Addressing these questions will further contribute to unifying conceptually the tools of manifold learning and topological data analysis.

References

1. He X, Cai D, & Niyogi P (2006) Laplacian score for feature selection. *Advances in neural information processing systems*, pp 507-514.
2. Hatcher A (2002) *Algebraic Topology* (Cambridge University Press).
3. Carlsson G (2009) Topology and data. *Bulletin of the American Mathematical Society* 46(2):255-308.
4. De Silva V & Ghrist R (2007) Coverage in sensor networks via persistent homology. *Algebraic & Geometric Topology* 7(1):339-358.

5. Muhammad A & Egerstedt M (2006) Control using higher order Laplacians in network topologies. *Proc. of 17th International Symposium on Mathematical Theory of Networks and Systems*, (Citeseer), pp 1024-1038.
6. Horak D & Jost J (2013) Spectra of combinatorial Laplace operators on simplicial complexes. *Advances in Mathematics* 244:303-336.
7. Belkin M & Niyogi P (2002) Laplacian eigenmaps and spectral techniques for embedding and clustering. *Advances in neural information processing systems*, pp 585-591.
8. Coifman RR*, et al.* (2005) Geometric diffusions as a tool for harmonic analysis and structure definition of data: multiscale methods. *Proceedings of the National Academy of Sciences of the United States of America* 102(21):7432-7437.
9. Parzanchevski O & Rosenthal R (2017) Simplicial complexes: spectrum, homology and random walks. *Random Structures & Algorithms* 50(2):225-261.
10. Mukherjee S & Steenbergen J (2016) Random walks on simplicial complexes and harmonics. *Random structures & algorithms* 49(2):379-405.
11. De Silva V, Morozov D, & Vejdemo-Johansson M (2011) Persistent cohomology and circular coordinates. *Discrete & Computational Geometry* 45(4):737-759.
