# Supplementary figures and images for: Clustering-independent analysis of genomic data using spectral simplicial theory
Source: PLoS Comput Biol. 2019 Nov 22;15(11):e1007509. doi: 10.1371/journal.pcbi.1007509 (PMC6897424; doi:10.1371/journal.pcbi.1007509)

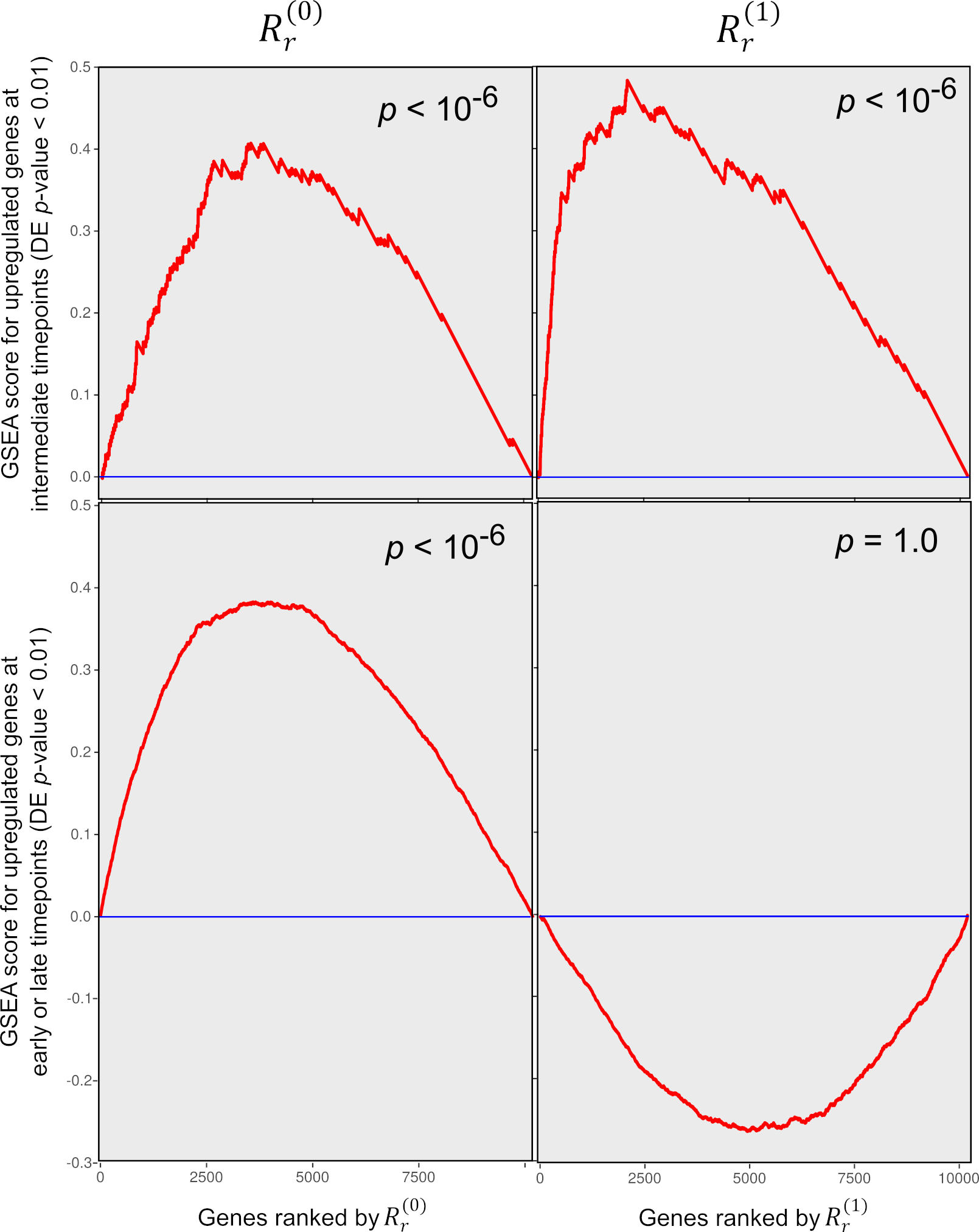

Supplement: S1 Fig — Gene-set enrichment analysis for upregulated genes at intermediate (top) or early/late (bottom) states in the example of the in vitro differentiation of mESCs into MNs using standard and direct programming protocols. Genes are ranked according to their 0- (left) and 1-dimensional (right) combinatorial Laplacian score. Genes with 0-dimensional combinatorial Laplacian score are enriched for genes that are differentially expressed at any stage within the differentiation. Genes with low 1-dimensional combinatorial Laplacian score are strongly enriched for genes that are upregulated at intermediate states, where the alternative paths for differentiation occur. (TIF) [file pcbi.1007509.s002.tif]

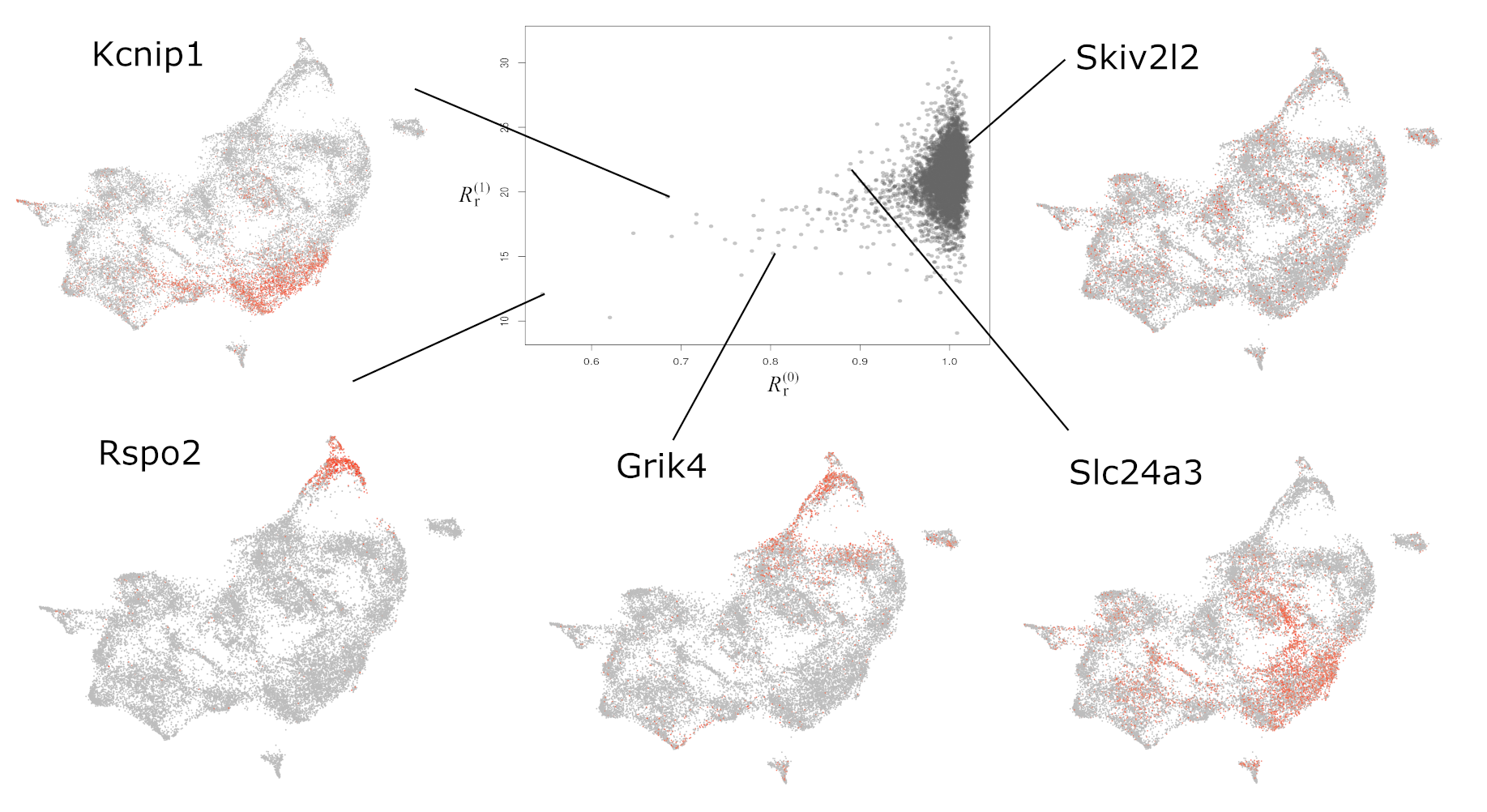

Supplement: S2 Fig — The scatter plot represents the 0- and 1- dimensional combinatorial Laplacian scores of 6,691 genes. A UMAP representation color-coded for expression (grey to red) is shown for some of the top differentially expressed genes identified by this method. Genes with low values of Rr(1) have upregulated expression along the AER trajectory. For reference, the expression of a gene with high 0- and 1- combinatorial Laplacian scores (Skiv2l2) is also displayed. (TIF) [file pcbi.1007509.s003.tif]

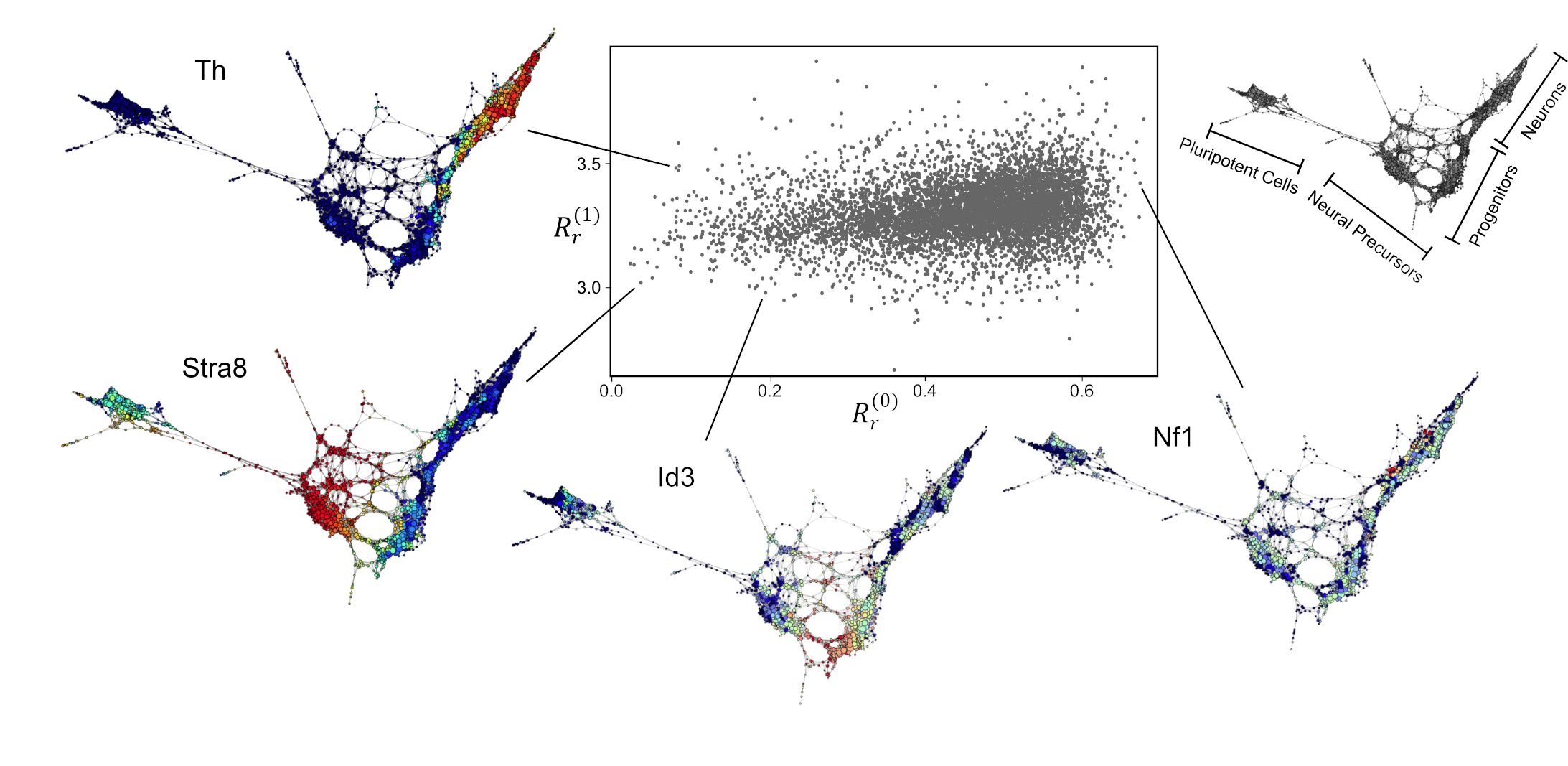

Supplement: S3 Fig — The 0- and 1-dimensional combinatorial Laplacian scores were run over the scRNA-seq expression data of the differentiation of mESCs into MNs using the SP protocol. The scatter plot represents the 0- and 1-dimensional combinatorial Laplacian scores of 6,938 genes. A Mapper simplicial complex color-coded for expression (blue: low expression; red: high expression) is shown for some of the top differentially expressed genes identified by this method. Genes with low values of Rr(1) have upregulated expression along the loops in the region of the neural precursors, where cell cycle effects are large. For reference, the expression of a gene with high 0- and 1- combinatorial Laplacian scores (Nf1) is also displayed. (TIF) [file pcbi.1007509.s004.tif]
